# Nutrient Intake during Pregnancy and Post-Partum: ECLIPSES Study

**DOI:** 10.3390/nu12051325

**Published:** 2020-05-07

**Authors:** Estefania Aparicio, Cristina Jardí, Cristina Bedmar, Meritxell Pallejà, Josep Basora, Victoria Arija, the ECLIPSES Study Group

**Affiliations:** 1 Nutrition and Public Health Unit. Research Group on Nutrition and Mental Health (NUTRISAM), Faculty of Medicine and Health Science, Universitat Rovira i Virgili, 43201 Reus, Spain; estefania.aparicio@urv.cat (E.A.); cristina.jardi@urv.cat (C.J.); cristina.bedmar@urv.cat (C.B.); 2 Pere Virgili Institute for Health Research (IISPV), Universitat Rovira i Virgili, 43003 Tarragona, Spain; 3 Tarragona-Reus Research Support Unit, Jordi Gol Primary Care Research Institute, 43003 Tarragona, Spain; meritxell.palleja@urv.cat (M.P.); jbasora.tarte.ics@gencat.cat (J.B.); 4 CIBERobn (Center for Biomedical Research in Physiopathology of Obesity and Nutrition), Instituto de Salud Carlos III, 28029 Madrid, Spain

**Keywords:** pregnancy, lactation, post-partum, energy and nutrient intake, adequacy

## Abstract

Pregnancy and post-partum are critical periods in which nutritional intake is essential to maternal and child health. Our aim was to describe dietary intake during pregnancy and post-partum and assess its adequacy. A longitudinal study was conducted on 793 pregnant women. Data about maternal characteristics, health, diet and lifestyle were assessed. Energy and nutritional intake were compared to the Recommended Dietary Allowances (RDA). The results showed that the intake of energy (82.6%), protein (80.6%) and carbohydrate (99.5%) was adequate (above 80% of RDA) during pregnancy, as were vitamins C, B2 and B12; but vitamin D, iron and folate intake were a long way from RDA (below 35%). Similar results were observed for the post-partum period although fiber, and vitamins E and C decreased compared to intake during pregnancy. In conclusion, although nutritional requirements increase during gestation, pregnant women did not increase their energy and nutritional intake during pregnancy and postpartum and they had a high risk of deficient intake of vitamin D, iron and folates during pregnancy, and therefore, of developing an unfavorable nutritional status, contrary to health recommendations. These findings underscore the necessity of intensive nutrition programs during and after pregnancy.

## 1. Introduction

Pregnancy and lactation are an essential stage of the lifecycle during which an adequate diet is crucial in order to meet the increased nutritional requirements of the mother [1], respond to the physiological demands of pregnancy and milk production, and ensure the healthy development of the fetus [2]. 

Inadequate maternal nutrition, and especially a deficit of essential nutrients, is associated with negative health outcomes in both the mother and the child [3,4,5,6]. The key nutrients that are particularly important during pregnancy and lactation include iron, folates, calcium and vitamin D [7,8]. Poor maternal nutrition is associated with iron deficiency, which can lead to low birth weight (<2500 g); folate deficiency, which can cause neural tube defects [9,10]; and calcium deficiency, which increases the risk of hypertension during pregnancy and also contributes to bone demineralization [11]. Vitamin D plays an important role in bone metabolism through the regulation of calcium homeostasis. Vitamin D deficiency has been linked to an increased risk of preeclampsia, gestational diabetes mellitus, and other specific conditions of certain tissues in pregnant women [5,12,13,14,15].Additionally, recent studies and meta-analyses have revealed other health repercussions including adverse anthropometric outcomes, preterm birth, small for gestational age and adverse neurodevelopmental outcomes in childhood [16,17,18]. 

Consequently, a high-quality diet, such as the healthy eating pattern of the Mediterranean diet, is internationally recommended for pregnant and breastfeeding women along with an increased caloric and specific nutrient intake, depending on the pregnancy trimester [1]. Nevertheless, several studies on a national or international scale have shown that, in general, pregnant women do not follow the recommended healthy diet [19,20,21,22,23,24,25,26], which could contribute to a suboptimal intake of key nutrients essential to the health of the mother and child. Indeed, some research in developed countries has found that a high percentage of pregnant women have a below-recommended intake of iron, calcium, vitamin D, and folates [19,21,27,28,29,30]. Recently, a study conducted in Spain found a lower-than-recommended intake of carbohydrates, n-3 and n-6 fatty acids, iron, folates and vitamin D [21], although it only evaluated the maternal diet during the first trimester. Another study in Spain assessed food consumption during pregnancy and lactation, but did not analyze nutritional intake [22]. To our knowledge, very few studies have been conducted recently in developed countries that assess nutritional intake during all trimesters of pregnancy [27,31] and during lactation [32].

Due to the importance of maternal nutrition, determining habitual nutrient intake throughout pregnancy and even after delivery is crucial. Therefore, the objective of our study is to describe nutritional intake during pregnancy and the post-partum period, and to assess its adequacy in healthy pregnant women from a European Mediterranean country.

## 2. Materials and Methods 

### 2.1. Study Design

A longitudinal study of pregnant women was carried out with follow-up from the first trimester of gestation to the post-partum period. The data were obtained from the ECLIPSES study, which is a randomized clinical trial aimed to evaluate the effectiveness of different doses of iron supplementation during pregnancy. The ECLIPSES trial was registered in ClinicalTrials.gov with identification number NCT03196882 and in the EU Clinical Trial Register, EUCTR-2012-005480-28. The Clinical Research Ethics Committee of the Jordi Gol Institute for Primary Care Research (IDIAP) and the Pere Virgili Institute for Health Research (IISPV) approved this study. We obtained informed consent from all pregnant women who agreed to participated in the study. Details of the protocol have been published previously [33].

### 2.2. Population

The participants were healthy pregnant women recruited by midwives from 12 sexual and reproductive health care services (ASSIR) of the Catalan Institute of Health (ICS) in Tarragona, Spain. Pregnant women were invited to participate in the study during their first prenatal visit with midwives and were included according to the following inclusion criteria: healthy adult woman over 18 years old, pregnancy at ≤ 12 weeks, capable of understanding the local and official languages in the region (Spanish or Catalan) and able to comprehend the requirements of the study, and sign the informed consent form. The exclusion criteria were as follows: multiple pregnancy, taking iron supplements (>10 mg) during previous months up until week 12, hypersensitivity to egg protein, previous serious illness (immunosuppression) or any chronic disease that could affect nutritional development (cancer, diabetes, malabsorption, or liver disease). 

The present study included all pregnant women in the intervention and control groups because our aim was to assess nutritional intake. Therefore, 793 pregnant women were included in the study during the first prenatal visit at week 12 of pregnancy. 

### 2.3. Data Collection

The data that was collected included the medical and obstetric history, socioeconomic information, lifestyle habits and anthropometric measurements in the first trimester of pregnancy (at the 12th week). Nutrition was assessed at the end of each trimester of pregnancy (at week 12, week 24 and week 36) and post-partum. Midwives and nutritionists compiled this information during the personal interview and from specific questionnaires.

The medical history and socioeconomic information collected included the following: maternal age, ethnicity, education level (primary, secondary, and university studies), estimated date of delivery, planned pregnancy, clinical and obstetric history. The socioeconomic level was calculated by occupational status using the Catalan classification of occupations (CCO-2011) [34] and was classified as low, middle, high.

Information about lifestyle habits was collected including alcohol intake, smoking habits and physical activity using the short version of the International Physical Activity Questionnaire (IPAQ-S) [35] and women were classified as sedentary or active.

Anthropometric measures were height (cm) and weight (kg). Body mass index was calculated and was classified following World Health Organization (WHO) criteria [36]: Normal weight (BMI <25 kg/m^2^) and excess weight (BMI ≥ 25 kg/m^2^).

#### Energy and Nutrient Intake 

Food consumption was assessed by a semi-quantitative food frequency questionnaire (FFQ) validated in our population [37]. The questionnaire and coding instructions can be found in the Supplementary Materials (Table S1). Participants completed the FFQ and reported their usual food consumption previous to week 12 (first trimester), at week 24 (second trimester), at week 36 (third trimester) and at 40 days post-partum. The FFQs were collected by specialized midwives, and nutritionists were in charge of reviewing, entering the food data and also the data scrubbing and analysis. The questionnaire contained 45 food groups and asked about the usual frequency of consumption per week or per month for food and beverages. The size and weight of a serving portion were standardized according to the validation questionnaire [37]. Each food item was calculated by grams per day. From this information we extracted energy intake (kcal/day), macronutrients (g/day) and micronutrients (mg or µg/day). We calculated the daily intake of energy and nutrients with the REGAL (Répertoire Général des Aliments) food composition table [38], which was complemented by the Mataix Verdú Spanish food composition table [39]. After that, we calculated the mean intake of all participants in each trimester of gestation and the mean of three trimesters to compare it with post-partum intake. We calculated the percentage of energy contributed by macronutrients to the total energy intake. As regards simple carbohydrates, natural sugars included whole fruits, vegetables, milk and cereals (rice, bread, pasta, flour), while free sugars included sweetened dairy desserts, sugary drinks (fresh and processed juices, soft drinks), sweetened cereals (sweetened breakfast cereals, biscuits, baked goods), chocolate, sugar and honey. We calculated the percentage of energy contributed by free sugar to the total energy intake. We compared the energy and nutrient intake found in our study with the recommended dietary allowance (RDA) for energy and each nutrient as outlined in the dietary reference intakes (DRI) recommended by the National Academy of Sciences from the US [40,41,42,43,44,45].

### 2.4. Statistical Analysis

The statistical analysis were run using SPSS version 20.0. The results were expressed as mean and standard deviation for quantitative variables or percentages for qualitative variables. To compare means of energy and nutrient intake was used paired t-test between two related groups. For all analyses, statistical significance was determined by a *p*-value of < 0.05. 

## 3. Results

A total of 793 pregnant women were enrolled in their 12th week of pregnancy, of which 395 were followed up at 40 days post-partum.

The baseline characteristics and mean daily energy and nutrient intake during the first trimester of pregnancy are presented in Table 1. The pregnant women had a mean age of 30.5 ± 4.9 years, 40.9% had completed at least secondary studies and 68.3% were middle class. About 15% smoked and consumed alcohol during pregnancy, 36.6% were categorized as being of excess weight and 92.9% were sedentary. More details on the characteristics of pregnant women can be found in the paper published on food intake from the same ECLIPSES study [20]. When the adequacy of nutritional intake was assessed according to the Institute of Medicine’s recommendations, it was found that pregnant women in their first trimester had an intake below 35% of RDA for iron (27.8%), vitamin D (11.7%), folate (33.5%) and below 70% of RDA for calcium (64.1%), vitamin B1 (64.8%), B6 (67.4%), E (66.3%). The intake of the rest of the nutrients was more acceptable and no pregnant women showed an intake above the upper limits of recommendation, except for niacin (there was only one woman).

When the mean daily energy and nutrient intake of the three trimesters was compared to the post-partum period (Table 2), a significantly lower intake of carbohydrates, natural sugars, free sugars, fiber, vitamin E and vitamin C was observed, as well as a significantly higher intake of monounsaturated fats, vitamin A, niacin, and vitamin B12 in post-partum women. When the adequacy was assessed with respect to the Institute of Medicine’s recommendations, adequate intake (above 80% of RDA) during pregnancy was found for energy (82.6%), protein (80.6%), carbohydrate (99.5%), vitamin C (88.7%), vitamin B2 (95.1%) and vitamin B12 (170.8%), however, the intakes were a long way from the RDA (below 35%) for vitamin D (12.3 %), iron (28.3 %) and folates (33.8%). Similar results were observed in the post-partum women, although fiber, vitamin E and C decreased compared to intake during pregnancy. Specifically, the percentage of iron varied from 28.3% to 84.4% of the RDA because the amount of iron recommended in pregnancy (27 mg/d) was much higher than during the post-partum period (9–10 mg/d). 

Regarding the average percentage of energy contributed by macronutrients in pregnancy, 12.9% was from proteins, 37.9% from carbohydrates (below the recommended range) and 49.2% from fats (above the recommended range). Moreover, the percentage of energy contributed by free sugar was 9%, and with respect to fatty acids, saturated fatty acids were above the recommended range (10%). In the post-partum period, the percentage of energy contributed by macronutrients was similar to pregnancy.

The nutrient intake through the three trimester of gestation and the postpartum is presented in Supplementary Materials, Tables S2 and S3. 

## 4. Discussion

Our study revealed that pregnant women in our region scarcely modify their nutritional intake during pregnancy and postpartum, despite the fact that an increase is recommended to cover their nutritional needs during this period, and which is critical for the health of the mother, fetus, and newborn child. Thus, although the intake of some nutrients is close to recommendation, such as energy, protein, carbohydrates, vitamin C, vitamin B2 and vitamin B12; other micronutrients are a long way from the RDA, such as vitamin D, folate and iron in pregnancy and vitamin D and folate in the postpartum period. 

Indeed, the scientific community has recently focused attention on the importance of adequate nutrition during the first one thousand days of life, including early pregnancy and lactation. For this reason, we analyzed the adequacy of dietary intake compared to current recommendations. Our results show that energy intake during the first trimester approached the recommendations. However, it failed to increase in keeping with the rise in caloric demand as the pregnancy progressed, as international organizations have recommended. Other studies in different settings have yielded similar results to ours, with energy intake remaining unchanged across trimesters [27,31,32]. This failure to achieve sufficient energy intake may be related to strict weight monitoring measures implemented by midwives and obstetricians. Furthermore, the traditional notion that pregnant women should “eat for two” has been reversed. As a result, the pregnant population has received the message to restrict their caloric intake, when it is more advisable to maintain an adequate caloric intake by following a healthy diet.

In terms of macronutrient intake, we found that the consumption of carbohydrates was lower and the fat intake was higher than recommended. The percentage of energy from fat was almost fifty percent, which is higher than the healthy diet recommendation (ranging from 30% to 35%). Previous studies conducted in our region [21,23] and elsewhere [27,30,46] found similar results. For instance, Rodriguez-Bernal et al. [21] in a sample of 822 pregnant Spanish women from the Mediterranean region documented a higher intake of fat, but omega 3 and omega 6 fatty acid intakes below the RDA. The research of Savards et al. [27] in a longitudinal study of 79 pregnant women, and Dubois et al. [30] in a cross-sectional study of 1533 participants, both from Canada, also found a high percentage of fat intake. In addition, although Saunder et al. [28] found a fat intake in the normal range in a large sample of 1674 Norwegian pregnant women, the consumption of saturated fatty acid was high. In this regard, our results also showed that the percentage of saturated fatty acids exceeded the recommendations (<10%) during pregnancy and post-partum. 

The recommended percentage of energy from carbohydrates of 50% to 55% was not achieved at any time during gestation in our sample. This finding is corroborated by a recent meta-analysis [9] and other research [28]. Aside from total amount, carbohydrate quality is also important. The consumption of whole grains, legumes, vegetables and whole fruits have a protective effect on health in contrast to refined grains and free or added sugar [47]. The low intake of carbohydrate that was observed coincides with limited consumption of grains, legumes, fruits and vegetables, as we reported in our previous study [20]. An increase would therefore be advisable. Notably, free sugar intake was almost 9%, just under the upper limit recommended by the WHO (less than 10% of the total energy value). We should underscore that 10% is the maximum value and that intakes below 5% have been associated with better health outcomes, such as a decreased incidence of dental cavities [48]. In fact, a diet rich in sugar during pregnancy can increase the risk of cavities in offspring at five years old by 1.5 times [49]. Pregnancy frequently involves changes in satiety and food choice, and especially in sugar intake, which is probably due to cravings for sweet foods during the first to the second trimester [24]. Additionally, our results showed that fiber intake was below the recommended values. Similar results have been noted by other authors [21,23,27]. Significantly, adequate fiber intake may prevent several pregnancy-related health concerns such as constipation and gestational diabetes [50]. 

Our findings also show that pregnant women are at risk of inadequate intake of several minerals and vitamins. In this study, the intake of folate and iron were a long way from the RDA (with a percentage below 35% RDA) They are also at risk of insufficient vitamin D intake with an adequacy of only around 12.3%, and insufficient calcium intake (the adequacy was only 67.2%). All of this highlights the risk related to the low consumption of fruit, vegetables, legumes, nuts, dairy products and poultry, fish and eggs, as was observed in our previous research [20]. Insufficient mineral and vitamin intake, especially folate, iron, vitamin D and calcium, are consistent with the findings of previous studies [19,21,27,28,29,30]. 

Folate is essential during the embryonic and fetal stages of pregnancy and a deficient status of folate increases the risk of preeclampsia and fetal anomalies, among other deleterious effects on child development [50]. Our study found a low intake of folate, that is, only 33.8% of the RDA. Likewise, other studies in developed countries have also revealed the insufficient intake of this micronutrient among pregnant women [19,21,27,28,29,30]. Consequently, the systematic supplementation of folic acid both before and during pregnancy is currently recommended [9]. However, studies conducted in Canadian [27] and American pregnant women [29] reported that women taking supplements exceeded the recommendations for folate, a result corroborated by Navarrete in a Spanish cohort [51]. The health consequences of high serum folate levels during pregnancy are unknown and should be investigated. 

For iron, the IOM recommends an increase from 18 mg/day (adult female) to 27 mg/day for pregnant women. In our sample, iron intake reached only 27.8% of the RDA, with a mean dietary intake of 7.7 mg/d. Similar low intakes (around 30%) have been reported in Spain and other European studies in pregnant women [21,23,52]. Iron deficiency anemia in pregnancy is related to preterm birth, low birth weight [50] and impaired psychomotor development and cognitive function in the child [53]. However, appropriate iron supplementation must be studied, as women can exceed the upper limit intake when both iron from food and from the supplement are taken into consideration [28,54]. High dosages of iron may also be harmful to child neurodevelopment [53]. In fact, a recent clinical trial in Spain conducted by our research team found that iron supplementation should be adapted individually to early pregnancy levels of hemoglobin and iron stores [55]. This study found high percentages of women, ranging from 7.39% to 11.9%, with iron deficiency anemia, and 6.8% to 13.1% of the pregnant women in our study were found to be at risk of hemoconcentration (hemoglobin >130 g/L [55].

In addition to iron and folate, the importance of maintaining adequate levels of vitamin D cannot be ignored, as its deficiency has been linked to adverse maternal and child health outcomes [18]. Our results revealed a mean intake of 1.8 µg/day in the period of gestation and post-partum, only about 12.3% and 11.7% of the RDA (15 µg/day), respectively. Other researchers have also found high rates of inadequate vitamin D intake, even when subjects taking supplements were included [21,27]. A systematic review recently concluded that there is a moderate association between adequate maternal levels of vitamin D and bone health in the child [56], but more evidence is needed before using supplements can be recommended as a means to prevent undesirable health outcomes [50]. The consumption of food rich in vitamin D together with sufficient sun exposure could be encouraged during pregnancy and lactation while monitoring plasma levels of vitamin D at the same time in order to prescribe supplements if needed. 

Calcium intake is essential during gestation for appropriate bone development in the fetus and also during lactation to maintain good maternal health. Moreover, an adequate intake of calcium, along with vitamin D, is necessary for a proper bone mineralization. However, our findings showed that the calcium intake of pregnant women does not reach an adequate level. In our sample, the mean calcium intake was less than 70% of RDA (668.3 mg/day, SD 256.1) in the first trimester. Saunders et al. [28] also found a substantial percentage of women at risk for inadequate calcium intake. In contrast, one meta-analysis revealed a calcium intake above the RDA [9], probably due to differences in dietary patterns from country to country. The consumption of dairy products, the best sources of dietary calcium, has been found to be quite low in pregnant women [20,22]. This deficiency in calcium intake increases the risk of preeclampsia, affects bone health, muscle contraction and child health outcomes [50]. 

In Europe, thiamine intake has been documented at 1.4 times the recommendation [9]. Nevertheless, our results showed that thiamine intake was below 70% of RDA, which may be a result of the lower intake of carbohydrates. Thiamine acts as a coenzyme and is involved in the metabolism of carbohydrates and fats. Thiamine deficiency can affect fetal brain development [50]. In regard to other micronutrients, we found that vitamin B6 and E intake were below 70% of the recommended level, as other research has also found [19,21,23,28,29]. For instance, Arija et al. [19] found an increase in the inadequate intake of vitamin B6 at twenty-six weeks of gestation. Moreover, Rodriguez-Bernal et al. [21] showed that 67.8% of pregnant women had inadequate intake of vitamin E. Therefore, pregnant women should be advised to eat a greater proportion of fruit, vegetables, whole grains, seed and nuts.

The post-partum and lactation periods are as important to maternal and child health as the gestation period. Few studies have assessed dietary intake after pregnancy [19,22,26,32]. Our findings show that women did not change their energy intake from pregnancy to 40 days post-partum, although there was an increase in fat and a decrease in carbohydrate intake (natural sugars and free sugars). This last finding is similar to that of another study that assessed the diet of 32 healthy women at six weeks post-partum [32]. We documented a post-partum decrease in the intake of fiber, and vitamins C and E, which is most likely attributable to the below-recommendation consumption of fruit, vegetables, nuts and grains, as Cuervo et al. [22] found in a large sample of pregnant and breastfeeding Spanish women. However, unlike our study, the work of Cuervo et al. did not assess nutritional intake. We found that the ingestion of other key nutrients (mainly folate, vitamin D, B1 and calcium), which were below recommended levels during pregnancy, did not increase after delivery. In agreement with our results, the only previous research in our region, which was conducted in 2004 showed a decrease in nutrient intake at six months post-partum, and an inadequate intake of calcium, folate and vitamin B6 [19]. Remarkably, although the iron intake did not change from pregnancy to post-partum, its percentage of adequacy increased during post-partum to around 84%. This is due to the recommendation that iron should be decreased from 27 mg/day (pregnant woman) to 9 mg/day (lactation woman).

Overall, in spite of the fact that an increase energy and nutrient intake during pregnancy and lactation is advisable, pregnant women ingested low amounts of certain micronutrients, mainly iron, folate and vitamin D. As a result, they did not meet the RDA for key nutrients, which can adversely affect the health of both mother and child. Therefore, we believe that individualized dietary advice should be offered before and during pregnancy as well as after delivery in order to ensure that energy requirements are met and a balanced nutritional intake is achieved, especially in relation to micronutrients. This is important in order to attain satisfactory nutritional status among pregnant women and to achieve optimal fetal development while not exceeding appropriate maternal weight gain during gestation. 

### Strengths and Limitations

This study contributes to the body of knowledge on dietary intake in pregnancy and the post-partum period in Spain for the purpose of determining possible deficiencies and taking preventive measures. The strengths of this study are noteworthy: We used a longitudinal design and a large sample size; we assessed nutrient intake with a semi-quantitative FFQ validated in our population, which was easy to administer and provided data on energy and nutrient intake [37]; and, lastly, we calculated the adequacy of the nutrient intake compared to the standard recommendation. Nevertheless, our study also has some limitations. For instance, the amount of complete dietary data decreased from the pregnancy to the post-partum period, and potential subjects who were taking iron supplements were excluded due to the original design of the clinical trial. 

In summary, despite evidence of the importance of gestational nutrition for the health of both mother and child, our findings show that nutrient intake during pregnancy does not reach the recommended levels for critical nutrients in pregnancy and during the post-partum period. Therefore, intensive dietary advice is needed during pregnancy and the post-partum period to ensure that mothers follow an adequate nutritional program. Improving the nutritional status of mothers from early pregnancy, or even before, and after delivery may prevent critical nutrient deficiencies and can benefit the health of both the mothers and their children. More research is needed for a more in-depth assessment of dietary intake, and nutritional interventions should be carried out in these stages of the lifecycle.

## 5. Conclusions

Although nutritional requirements increase during gestation, pregnant women from a European Mediterranean country tend to maintain their caloric intake. The intake during pregnancy was close to the recommendations for energy, and important nutrients, such as protein, carbohydrates, vitamin C, B2 and B12. However, there was a high risk of inadequate intake of essential nutrients, such as vitamin D, folates and iron. Intake was similar in the post-partum period, although fiber, and vitamins E and C decreased compared to pregnancy, and iron intake was adequate. Higher risk intakes need to be prevented through nutrition education programs before and after pregnancy so that mothers and children can avoid negative health outcomes. More research is needed for a more in-depth assessment of dietary intake, and nutritional interventions should be carried out in these stages of the lifecycle. 

## Figures and Tables

**Table 1:** Baseline characteristics and mean daily energy and nutrient intake and adequacy as a percentage of the recommended dietary allowances during the first trimester of pregnancy.

	First Trimester (*n* = 793)	
**General Characteristics**		
**Maternal age (years)**		30.5 (4.9)
**Maternal educational level (%)**	Primary studies	31.4 (249)
	Secondary studies	40.9 (324)
	University studies	27.7 (220)
**Social class (%)**	Low	16.0 (127)
	Middle	68.3 (542)
	High	15.6 (124)
**BMI (kg/m^2^)**		24.8 (4.5)
**Energy and nutrient intake**	**Intake**	**Adequacy percentage of the RDA**
	**Mean (SD)**	**Mean (SD)**
**Energy (Kcal/d)**	1755.7 (408.2)	88.4 (20.6)
**Proteins (g/d)**	56.1 (16.8)	78.9 (23.7)
**Carbohydrates (g/d)**	171.6 (64.9)	98.1 (37.1)
**Natural Sugars (g/d)**	20.2 (9.5)	ND
**Free Sugars (g/d)**	41.4 (28.5)	ND
**Lipids (g/d)**	93.2 (12.8)	ND
**Saturated fats (g)**	23.9 (5.8)	ND
**Monounsaturated fats (g)**	52.9 (4.6)	ND
**Polyunsaturated fats (g)**	9.9 (1.4)	ND
**Cholesterol (mg)**	219.4 (75.1)	ND
**Fiber (g/d)**	12.5 (4.5)	44.6 (16.1)
**Calcium (mg/d)**	668.3 (256.1)	64.1 (27.1)
**Iron (mg/d)**	7.7 (2.5)	27.8 (9.5)
**Vitamin A (μg/d)**	613.5 (189.1)	79.8 (23.8)
**Vitamin E (mg/d)**	9.9 (1.1)	66.3 (7.6)
**Vitamin C (mg/d)**	77.2 (34.8)	87.6 (42.3)
**Vitamin D (μg/d)**	1.8 (1.1)	11.7 (6.9)
**Vitamin B1 (mg/d)**	0.9 (0.3)	64.8 (21.2)
**Vitamin B2 (mg/d)**	1.3 (0.4)	91.8 (32.6)
**Niacin (mg/d)**	13.1 (4.3)	70.3 (23.7)
**Vitamin B6 (mg/d)**	1.3 (0.4)	67.4 (23.2)
**Vitamin B12 (μg/d)**	4.3 (1.5)	164.5 (59.1)
**Folate (μg/d)**	201.0 (72.9)	33.5 (12.2)

Values expressed as mean and standard deviation (SD) or %. Natural Sugars include: sugars from fruits, vegetables, milk and salted cereals; Free Sugars include: monosaccharides and disaccharides added to foods and beverages and sugars naturally present in honey, syrups, fruit juices and fruit juice concentrates; RDA: recommended dietary allowances. Data from Dietary Reference Intakes (DRIs) of the US Institute of Medicine for pregnant women; Dietary Reference Intakes for Calcium, Phosphorus (1997); Energy, Carbohydrates, Fiber, Lipids and Protein (2002/2005); Thiamine, Riboflavin, Niacin, Vitamin B6, Folates, Vitamin B12, (1998); Vitamin C, Vitamin E, (2000); Vitamin A, Iodine, Iron, (2001); Sodium, (2005); calcium and Vitamin D (2011);. ND, not determined. Vitamin A (μg/d): Calculated as retinol activity equivalents.

**Table 2:** Mean daily energy and nutrient intake and adequacy as a percentage of the recommended dietary allowances during pregnancy and post-partum.

	Comparison of Mean Intake and Adequacy for Three Trimesters versus Post-Partum				
Energy and Nutrient Intake	Three Trimesters ^a^ *n* = 395	% Adequacy Compared to the RDA	Post-Partum ^b^ *n* = 395	% Adequacy Compared to the RDA	*p*-Value ^(a-b)^
	Mean (SD)	Mean (SD)	Mean (SD)	Mean (SD)	
**Energy (kcal/d)**	1779.1 (356.0)	82.6 (19.6)	1744.8 (382.8)	70.8 (16.6)	0.282
**Proteins (%)**	12.9 (1.5)	ND	13.2 (1.9)	ND	0.001
**Carbohydrates (%)**	37.9 (5.5)	ND	36.8 (6.3)	ND	<0.001
**Natural Sugars (%)**	4.7 (1.5)	ND	4.3 (1.7)	ND	<0.001
**Free Sugars (%)**	9.0 (4.1)	ND	8.5 (4.8)	ND	0.028
**Lipids (%)**	49.2 (5.1)	ND	50.1 (5.9)	ND	0.001
**Saturated fats (%)**	12.6 (1.3)	ND	12.8 (1.5)	ND	0.001
**Monounsaturated fats (%)**	28.0 (3.6)	ND	28.6 (4.3)	ND	0.004
**Polyunsaturated fats (%)**	5.2 (0.6)	ND	5.3 (0.7)	ND	0.016
**Proteins (g/d)**	57.0 (13.6)	80.6 (20.0)	57.8 (16.6)	81.2 (23.2)	0.067
**Carbohydrates (g/d)**	171.7 (55.2)	99.5 (33.1)	164.8 (60.2)	77.9 (28.5)	0.009
**Natural Sugars (g/d)**	20.7 (8.2)	ND	18.7 (8.7)	ND	<0.001
**Free Sugars (g/d)**	42.2 (24.7)	ND	39.9 (28.4)	ND	0.011
**Lipids (g/d)**	94.3 (10.7)	ND	95.0 (12.7)	ND	0.080
**Saturated fats (g)**	24.4 (4.8)	ND	24.7 (5.7)	ND	0.285
**Monounsaturated fats (g)**	53.3 (3.9)	ND	53.8 (4.7)	ND	0.005
**Polyunsaturated fats (g)**	10 (1.2)	ND	10 (1.4)	ND	0.113
**Cholesterol (mg)**	219.7 (60.8)	ND	223.7 (73.0)	ND	0.052
**Fiber (g/d)**	12.5 (3.8)	44.8 (13.9)	11.9 (3.9)	40.7 (13.7)	0.004
**Calcium (mg/d)**	668.4 (208.0)	67.2 (22.8)	650.7 (254.4)	64.8 (25.2)	0.076
**Iron (mg/d)**	7.6 (2.2)	28.3 (8.2)	7.6 (2.3)	84.4 (25.8)	0.171
**Vitamin A (μg/d)**	611.6 (162.2)	77.4 (22.3)	659.1 (202.9)	83.5 (34.3)	<0.001
**Vitamin E (mg/d)**	10.0 (0.9)	66.7 (6.5)	9.8 (1.1)	51.7 (5.6)	<0.001
**Vitamin C (mg/d)**	75.9 (31.4)	88.7 (37.1)	67.8 (32.0)	56.0 (26.6)	<0.001
**Vitamin D (μg/d)**	1.8 (0.9)	12.3 (6.1)	1.8 (1.0)	11.7 (6.6)	0.076
**Vitamin B1 (mg/d)**	0.9 (0.2)	65.9 (17.8)	0.9 (0.3)	65.1 (19.4)	0.851
**Vitamin B2 (mg/d)**	1.3 (0.4)	95.1 (27.7)	1.3 (0.4)	83.4 (26.8)	0.327
**Niacin (mg/d)**	12.8 (3.6)	71.6 (19.8)	13.1 (4.3)	77.1 (24.9)	0.013
**Vitamin B6 (mg/d)**	1.3 (0.4)	68.4 (19.3)	1.3 (0.4)	63.3 (19.6)	0.505
**Vitamin B12 (μg/d)**	4.4 (1.3)	170.8 (50.9)	5.1 (1.8)	180.3 (64.5)	<0.001
**Folate (μg/d)**	204.0 (61.1)	33.8 (10.2)	196.7 (60.9)	39.2 (12.2)	0.065

Values expressed as mean and standard deviation (SD). Natural Sugars include sugars from fruits, vegetables, milk and salted cereals; Free Sugars include monosaccharides and disaccharides added to foods and beverages and sugars naturally present in honey, syrups, fruit juices and fruit juice concentrates. RDA recommended dietary allowances. Data from DRIs of the US Institute of Medicine for pregnant women; Dietary Reference Intakes for Calcium, Phosphorus (1997); Energy, Carbohydrates, Fiber, Lipids and Protein (2002/2005); Thiamine, Riboflavin, Niacin, Vitamin B6, Folates, Vitamin B12, (1998); Vitamin C, Vitamin E, (2000); Vitamin A, Iodine, Iron, (2001); Sodium, (2005); calcium and Vitamin D (2011);. ND, not determined. Vitamin A (μg/d): Calculated as retinol activity equivalents. ^a^ Three trimesters. ^b^ Post-partum.

## Supplementary Materials

The following are available online at https://www.mdpi.com/2072-6643/12/5/1325/s1, Table S1: Food frequency questionnaire and coding instructions. Table S2: Mean daily energy and nutrient intake during pregnancy. Table S3: Energy intake and percentage of total energy contributed by macronutrients during pregnancy and post-partum.
