# Supplementary material for: Nutrient Intake during Pregnancy and Post-Partum: ECLIPSES Study
Source: Nutrients. 2020 May 7;12(5):1325. doi: 10.3390/nu12051325 (PMC7285175; doi:10.3390/nu12051325)
Supplement: Supplementary file 1 [file nutrients-12-01325-s001.pdf]

Supplementary Materials:

Table S1. Food frequency questionnaire and coding instructions.

Table S2. Mean daily energy and nutrient intake during pregnancy

Table S3. Energy intake and percentage of total energy contributed by macronutrients during pregnancy and post-partum

Table S1. Food frequency questionnaire and coding instructions

EATING HABITS

The following questionnaires ask you about your child's eating habits and frequency of food consumption.

A) Eating habits

Choose the answer according to your child's habits:

1. What kind of milk or vegetable drink do you usually drink?	1. Cow milk	2. Soy drink	3.Other (which one)
2. What kind of cow's milk do you usually drink?	1. Whole milk	2. Semi-skimmed milk	3. Skimmed milk
3. Which kind of yogurt do you usually eat?	1. Whole yogurt	2. Semi-skimmed yogurt	3. Skimmed yogurt
4. Do you usually sweeten the food (yogurt, milk, fruit...) with sugar, honey, sweetener…? Do you add sugary cocoa like *Nesquik*?	1. No	2. Yes (number of servings) _________	
5. What kind of bread do you eat?	1.White bread	2. Whole grain bread	3. Other (which one)
6. What kind of oil do you use to dress salads?	1. Olive oil	2. Seed oil (sunflower, corn, …)	3. olive oil or seed oil
7. What kind of oil do you use for cooking?	1. Olive oil	2. Seed oil (sunflower, corn, …)	3. olive oil or seed oil
8. How much oil do you use to cook?	1. Plenty	2. Moderate	3. Little
9. At the eating moment, do you add more oil to your dishes (example: salads, …)?	1. Usually	2. Sometimes	3.Ocasionally
10. Do you add to bread or sandwiches?	1. Usually	2. Sometimes	3.Ocasionally
11. Do you add butter to bread or sandwiches?	1. Usually	2. Sometimes	3.Ocasionally

B) Food frequency questionnaire

The following questionnaire asks about the frequency in which your child consumes**. The frequency of consumption must be specified in the boxes found on the right side of each food.**

For each food item in the list, you should write down the number of times your child consumes it:
• If your child eats it every day of the week (once a day), put a 7 in the WEEKLY column.
• If your child consumes it once a week, put the times: 1-2-3-4-5 or 6 in the column WEEKLY.

Always think about adding up the consumption of all the meals of the day (breakfast, lunch, snack, dinner, others ...)

For example:
- If your child drinks milk for breakfast every morning and occasionally before go to bed too: 7 + 3 = 10 times a week.
- If your child takes meat 4 times for lunch and 2 times for dinner: 4 +2 =6 times a week.

Only check one box for each food (WEEKLY or MONTHLY)
• If your child consumes food ones a month, write down how many times. 1-2-3 ... in the MONTHLY column
• If your child never or almost never, leave the blank box, without putting anything in it.

**FOODS LIST**	**HOW MANY TIMES...?**	
	**WEEKLY**	**MONTHLY**
Milk	*11*	
Muffin	*7*	
…		
Fish		*3*

Example: If your child usually drinks milk for breakfast (7 times a week) and muffins (7 times a week) and sometimes drinks milk before going to bed (4 times). Moreover, if your child eats 3 times a month (3 times).

Food frequency questionnaire – FFQ

HOW MANY TIMES DO YOU EAT/DRINK---?		
	**WEEKLY**	**MONTHLY**
Milk		
Yogurt		
Chocolate: chocolate bar, *Kit-Kat, Mars Chocolate Brands*...		
Breakfast Puffed Cereals *(Corn-Flakes, Kellogg's, Quaker…)*		
"Maria" Cookies		
Chocolate cookies, cream cookies...		
Muffins, soft cakes, biscuits		
Ensaimada or spiral-shaped pastry, Donut, Croissant		
Salad: lettuce, tomatoes, curly endive....		
Green bean, chard, spinach…		
Garnish Vegetables: eggplant, zucchini, mushroom…		
Potatoes: baked, fried or boiled		
Legumes: lentils, peas, chickpeas, beans…		
White rice, paella		
Pasta: noodles, macaroni, spaghetti…		
Soups and creams		
Eggs		
Chicken or turkey		
Beef, pork, lamb (steak, battered…)		
Minced meat: pork sausage, hamburger, meatballs…		
White fish: hake, grouper...		
Blue fish: sardine, tuna, salmon....		
Seafood: mussels, prawn, octopus, squid…		
Croquettes, small pasty, pizza		
Bread (sandwich, bread consumed at meals)		
Serrano ham, ham, cold meat		
White or fresh cheese (*Burgos*,...) or low fat cheese		
Other cheeses cured or semi-cured, creamy…		
Citrus fruit: orange, mandarin orange…		
Other fruits: Apple, pear, peach, apricot, banana…		

Canned fruit (in syrup)		
Natural fruit juice		
Commercial fruit juices		
Nuts: peanuts, hazelnuts, almonds…		
Dairy desserts: custard, crème caramel, junket,…		
Cream and chocolate cakes		
Snack bags (chips, *Cheetos*, fries...)		
Candies: candies, jelly beans…		
Ice creams: in summer/ in winter		
Sugary drinks (*Coca-Cola*, *Fanta*, Ice tea...)		
Low calorie drinks (*Coca-Cola* light, cero...)		

Table S2. Mean daily energy and nutrient intake during pregnancy

	Comparison intake first trimester versus second trimester			Comparison intake second trimester versus third trimester		
	First trimester[a] n=513 Mean (SD)	Second trimester[b] n=513 Mean (SD)	p-value between (a-b)	Second trimester[b] n=426 Mean (SD)	Third trimester[c] n=426 Mean (SD)	p-value between (b-c)
**Energy (Kcal/d)**	1794.5 (414.9)	1787.9 (389.6)	0.819	1788.5 (382.2)	1740.9 (372.5)	0.003
**Proteins (g/d)**	57.9 (17.2)	57.9 (15.4)	0.862	58 (15.7)	56.5 (15.9)	0.029
**Carbohydrates (g/d)**	177.2 (65.7)	173.9 (63.2)	0.362	173.9 (60.5)	165.9 (59.6)	0.002
**Natural Sugars (g/d)**	21.5 (9.7)	20.8 (9.4)	0.265	20.8 (9.4)	20.8 (9.9)	0.794
**Free Sugars (g/d)**	42.5 (28.8)	45.1 (30.1)	0.006	45.1 (30.1)	40.9 (27.6)	0.001
**Lipids (g/d)**	94.5 (12.9)	95.2 (12.4)	0.261	95.3 (12.5)	94.1 (12.1)	0.051
**Cholesterol (mg)**	219.7 (75.8)	225.4 (70.1)	0.053	225.6 (71.1)	220.6 (69.6)	0.353
**Saturated fats (g)**	24.4 (5.8)	24.9 (5.6)	0.058	25.0 (5.6)	24.5 (5.5)	0.074
**Monounsaturated fats (g)**	53.4 (4.7)	53.6 (4.6)	0.334	53.6 (4.6)	53.2 (4.4)	0.051
**Polyunsaturated fats (g)**	10.1 (1.4)	10.0 (1.4)	0.531	10.0 (1.4)	9.9 (1.3)	0.014
**Fibre (g/d)**	13.0 (4.6)	12.5 (4.3)	0.002	12.5 (4.3)	12.0 (4.1)	0.003
**Sodium (mg/d)**	1352.2 (419.2)	1326.8 (408.9)	0.065	1325.4 (414.7)	1281.7 (401.9)	0.006
**Potasium (mg/d)**	2253.3 (703.7)	2243.6 (645.3)	0.798	2241.3 (642.7)	2181.5 (647.7)	0.009
**Phosphorus (mg/d)**	853.3 (263.1)	865.2 (236.3)	0.092	863.8 (237.2)	848.2 (242.8)	0.125
**Calcium (mg/d)**	674.6 (266.2)	687.6 (229.2)	0.039	686.6 (227.9)	680.3 (253.5)	0.599
**Iron (mg/d)**	7.8 (2.7)	7.7 (2.4)	0.908	7.7 (2.4)	7.41 (2.3)	0.003
**Vitamin A (µg/d)**	616.6 (199.6)	620.9 (188.1)	0.319	623.3 (188.7)	613.1 (189.1)	0.121
**Vitamin E (mg/d)**	10.1 (1.2)	10.1 (1.1)	0.226	10.1 (1.1)	9.9 (1.1)	0.058
**Vitamin C (mg/d)**	79.2 (37.4)	75.5 (36.0)	0.040	75.6 (36.1)	74.6 (36.3)	0.257
**Vitamin D (µg/d)**	1.8 (1.1)	1.9 (1.0)	0.278	1.9 (1.0)	1.8 (1.1)	0.308
**Vitamin B1 (mg/d)**	0.9 (0.3)	0.9 (0.3)	0.712	0.9 (0.3)	0.9 (0.3)	0.050
**Vitamin B2 (mg/d)**	1.3 (0.5)	1.4 (0.4)	0.055	1.4 (0.4)	1.3 (0.4)	0.726
**Niacin (mg/d)**	13.1 (4.5)	13.0 (4.0)	0.475	13.0 (4.1)	12.6 (3.9)	0.027
**Vitamin B6 (mg/d)**	1.3 (0.5)	1.3 (0.4)	0.309	1.3 (0.4)	1.3 (0.4)	0.027
**Vitamin B12 (µg/d)**	4.4 (1.6)	4.5 (1.5)	0.103	4.5 (1.5)	4.4 (1.6)	0.411

**Folate (μg/d)**	212.0 (76.5)	204.8 (69.4)	0.051	204.6 (69.8)	199.7 (65.7)	0.094

Values expressed as mean and standard deviation (SD). Natural sugar include: Sugars from fruits, vegetables, milk and salted cereals; Free sugar include: Monosaccharides and disaccharides added to foods and beverages and sugars naturally present in honey, syrups, fruit juices and fruit juice concentrates; Vitamin A (μg/d): Calculated as retinol activity equivalents.

Table S3. Energy intake and percentage of total energy contributed by macronutrients during pregnancy and post-partum

	Comparison intake first trimester versus second trimester			Comparison intake second trimester versus third trimester			Comparison intake third trimester versus post-partum		
	First trimester[a] n=513 Mean (SD)	Second trimester[b] n=513 Mean (SD)	p-value between (a-b)	Second trimester[b] n=426 Mean (SD)	Third trimester[c] n=426 Mean (SD)	p-value between (b-c)	Mean of three trimesters[c] n=395 Mean (SD)	Post-partum[d] n=395 Mean (SD)	p-value between (c-d)
**Energy (Kcal/d)**	1794.5 (414.9)	1787.9 (389.6)	0.695	1788.5 (382.2)	1740.9 (372.5)	0.001	1779.1 (356.0)	1744.8 (382.8)	0.282
**Proteins (%)**	12.8 (1.9)	12.9 (1.8)	0.295	12.9 (1.8)	12.9 (1.9)	0.807	12.9 (1.5)	13.2 (1.9)	0.001
**Carbohydrates (%)**	38.5 (6.5)	38.0 (6.1)	0.064	38.1 (6.0)	37.3 (6.2)	0.001	37.9 (5.5)	36.8 (6.3)	<0.001
**Natural Sugars (%)**	4.8 (1.8)	4.6 (1.8)	0.138	4.6 (1.9)	4.8 (1.9)	0.103	4.7 (1.5)	4.3 (1.7)	<0.001
**Free Sugars (%)**	9.0 (4.9)	9.6 (4.8)	0.006	9.5 (4.7)	8.9 (4.6)	0.003	9.0 (4.1)	8.5 (4.8)	0.028
**Lipids (%)**	48.6 (6.1)	49.0 (5.6)	0.097	48.5 (5.6)	49.8 (5.7)	0.001	49.2 (5.1)	50.1 (5.9)	0.001
**Saturated fats (%)**	12.4 (1.6)	12.6 (1.5)	<0.001	12.7 (1.6)	12.8 (1.5)	0.111	12.6 (1.3)	12.8 (1.5)	0.001
**Monounsaturated fats (%)**	27.7 (4.3)	27.8 (3.9)	0.513	27.8 (4.0)	28.4 (4.0)	<0.001	28.0 (3.6)	28.6 (4.3)	0.004
**Polyunsaturated fats (%)**	5.2 (0.6)	5.3 (0.6)	0.678	5.2 (0.6)	5.3 (0.6)	0.005	5.2 (0.6)	5.3 (0.7)	0.016

Values expressed as mean and standard deviation (SD). Natural sugars include: Sugars from fruits, vegetables, milk and salted cereals; Free Sugars include: Monosaccharides and disaccharides added to foods and beverages and sugars naturally present in honey, syrups, fruit juices and fruit juice concentrates.
